# Root-Associated Fungal Communities in Two Populations of the Fully Mycoheterotrophic Plant *Arachnitis uniflora* Phil. (Corsiaceae) in Southern Chile

**DOI:** 10.3390/microorganisms7120586

**Published:** 2019-11-20

**Authors:** Hector Herrera, Javiera Soto, Luz E. de Bashan, Inmaculada Sampedro, Cesar Arriagada

**Affiliations:** 1 Laboratorio de Biorremediación, Departamento de Ciencias Forestales, Facultad de Ciencias Agropecuarias y Forestales, Universidad de La Frontera, 01145 Temuco, Chile; hector.herrera@ufrontera.cl (H.H.); javiera.psp@gmail.com (J.S.); 2 The Bashan Institute of Science, 1730 Post Oak Court, Auburn, AL 36830, USA; luz@bashanfoundation.org; 3 Department of Entomology and Plant Pathology, 301 Funchess Hall, Auburn University, Auburn, AL 36849, USA; 4 Environmental Microbiology Group, Northwestern Center for Biological Research (CIBNOR), Calle IPN 195, 23096 La Paz, B.C.S., Mexico; 5 Departamento de Microbiología, Facultad de Farmacia, Universidad de Granada, 18071 Granada, Spain; isampedro@ugr.es

**Keywords:** endophytes, mycoheterotrophy, mycorrhizal fungi, organic acids, soil fungi, symbiosis

## Abstract

The microbiological interactions of the roots of non-photosynthetic plants in South America have been scarcely explored. This study analyzes culturable fungal diversity associated with the mycoheterotrophic plant *Arachnitis uniflora* Phil. (Corsiaceae) in southern Chile, growing in two different understoreys of native (*Nothofagus*-dominated) and mixed forest (native, *Cupressus* *sempervirens,* and *Pinus* *radiata*). Rhizospheric and endophytic fungi were isolated, cultured, and purified to identify microorganisms associated with *A. uniflora* roots. We showed the different fungi associated with the plant, and that these distributions are influenced by the sampling site. We isolated 410 fungal strains (144 endophytic and 266 from the rhizosphere). We identified 13 operative taxonomical units from plants sampled in the mixed forest, while 15 were from the native forest. Rhizospheric microorganisms were mainly related to *Penicillium* spp., whereas some pathogenic and saprophytic strains were more frequent inside the roots. Our results have also shown that the fungal strains are weak for phosphate solubilization, but other pathways such as organic acid exudation and indole acetic acid production can be considered as major mechanisms to stimulate plant growth. Our results point to new fungal associates of *A. uniflora* plants reported in Andean ecosystems, identifying new beneficial endophytic fungi associated with roots of this fully mycoheterotrophic plant.

## 1. Introduction

In spite of land plants being considered autotrophic, there are almost 400 fully mycoheterotrophic species distributed across 87 genera [1,2]. These plants completely depend on the carbon provided by a mycorrhizal fungus (as well as other mineral nutrients) throughout their life cycle [3]. They act as parasites on fungi with little benefits for the associated mycobionts [4,5,6]. Mycoheterotrophy is characteristic of non-photosynthetic plants, although some photosynthetic species may also show partial mycoheterotrophy, especially in the early developmental stages where seeds and plantlets do not have sufficient nutritional reserves to start germination and the development of plantlets [7,8]. Mycoheterotrophic plants require compatible mycorrhizal fungi which provide carbon to the associated plant. The degree of heterotrophy varies according to the nature of the carbon requirements of the plant (fully or partially mycoheterotrophic) and may comprise fungal endophytes, plant pathogenic fungi, saprophytic fungi, arbuscular- and ectomycorrhizal-forming fungi, which are efficient for nutrient uptake from diverse surrounding plants or decaying woods [9,10,11,12].

Chile has a diverse ecosystem and climatic conditions and possesses pristine ecosystems with little human intervention, especially in the south, where various centers of biodiversity of native plants can be found [13,14]. Among the native flora, the orchids comprising 72 species are partial mycoheterotrophs, especially in early developmental stages where the embryo depends on the carbon provided by a compatible mycorrhizal fungi to germinate its dust-like seeds and establish the plantlets, before turning partially or fully autotropic at plant maturity [2,15]. In Chile, full mycoheterotrophy is present in the achlorophyllous plant *Arachnitis uniflora* Phil. (Corsiaceae), which is distributed in different areas of Chile, Argentina, Bolivia, and the Falkland Islands [16]. Studies on this mycoheterotrophic plant have characterized arbuscular mycorrhizal fungi (AMF) as the main mycorrhizal association, and little information is available regarding symbiotic interactions with other fungal clades [17]. Ecological studies on this achlorophyllous plant have focused on characterizing root morphology and anatomy [18], molecular identification of AMF [17,19,20] and formation of mycorrhizal propagules in the tuberous roots [16]. The lack of information about ecology, symbiotic mechanisms of reproduction, and functional associations at the seedling stage is likely caused by the life cycle of this plant, which spends most of the time underground, mimetic with the environment, and a short flowering and fruiting season, making localization and collection difficult [18]. Therefore, it is important to characterize the adaptation mechanisms developed by this mycoheterotrophic plant to different habitats, and how the diversity of fungal symbionts may diverge according to different microhabitats.

Fungi living in the plant endosphere/rhizosphere can result in a positive effect with respect to plant growth, nutrient availability, pathogen control, and the support of several environmental stresses [21]. Such mechanisms are intensified in mycoheterotrophic plants by their dependence on the activity of fungi [22]. Fungal metabolites such as phytohormones, organic acids, and siderophores have been described as the main compounds produced by microorganisms to improve plant growth [23]. Such activities have been described in several microorganisms isolated from plants, but in mycoheterotrophic species these mechanisms have been scarcely explored.

The aims of this study were to analyze the culturable fungal diversity associated with *A. uniflora* roots colonizing two microhabitats in segments of the Coastal Mountains in southern Chile (the understorey of a native *Nothofagus*-dominated forest and the understorey of a mixed *Peumus boldus*, *Luma apiculata*, *Cupressus sempervirens*, and *Pinus radiata* forest) and to assess its activity for plant growth promotion. We hypothesized that fungal diversity in the rhizosphere and inside *A. uniflora* roots are not restricted to AMF and different fungal endophytes with plant growth promotion traits can be isolated from the rhizomes.

## 2. Materials and Methods 

### 2.1. Site Description and Sampling

Sampling of *A. uniflora* plants was carried out in a segment of the Coastal Mountains in Cholchol, Region of La Araucanía, southern Chile (October 2017). *A. uniflora* plants were found in two different microhabitats: sampling point 1, understorey of a native *Nothofagus*-dominated forest (38°34′10.5″ S 72°57′57.6″ W); and sampling point 2, understorey of a mixed *P. boldus*, *L. apiculata*, *C. sempervirens*, and *P. radiata* forest (38°34′09.9″ S 72°57′36.2″ W). Four random rhizomes of flowering plants from ten different populations at each sampling site were collected, placed in paper bags and immediately brought to the Bioremediation laboratory at the Universidad de La Frontera. Rhizosphere soil adhering to the rhizome was obtained to perform isolation of rhizosphere microorganisms. Furthermore, bulk soil samples were collected (0–20 cm deep) to perform a chemical characterization. 

### 2.2. Isolation and Characterization of Rhizospheric and Endophytic Fungi 

Isolation of endophytic and rhizospheric fungi was performed according to Blain et al. [24] with few modifications. To isolate rhizospheric microorganisms, the entire rhizome of four plants (<10 mm) from each sampling population and 500 mg of adhering soil were placed in a 500 mL Erlenmeyer flask containing 300 mL of phosphate saline buffer (PBS; 1.2 g L^−1^ K_2_HPO_4_, 0.18 g L^−1^ KH_2_PO_4_, 8.5 g L^−1^ NaCl) and placed in an orbital shaker for 45 min at 180 rpm. After that, the rhizospheric soil solutions were serial diluted into dilutions from 10^−1^ to 10^−5^ in sterile distilled water (1 mL of rhizospheric soil solution diluted in 9 mL of sterile distilled water). Then, 500 µL of the dilutions 10^−3^, 10^−4^, and 10^−5^ were plated in triplicate into Petri dishes containing 30 mL of modified potato dextrose agar (PDA; plus 100 mg L^−1^ streptomycin) and modified Murashige and Skoog (MS; 50% salt concentration) according to Faria et al. [25]. Plates were incubated at 25 ± 1 °C until individual strains were detected. Fungal strains were excised from the original plates and purified. All rhizospheric microorganisms were stored in PDA at 4 °C for further analyses.

Endophytic microorganisms were isolated after a superficial disinfection of the entire rhizome with a solution of 3 mL of sterile distilled water, 1 mL of sodium hypochlorite (5% chlorine), and 1 mL of 100% alcohol (for each 5 mL of solution) for 5 min, followed by five washes in sterile deionized water. An aliquot of 500 µL of the last wash was plated in PDA and MS media to rule out the presence of rhizospheric microorganisms in the samples. Four superficially sterile rhizomes (<10 mm) per sampled population were suspended in 50 mL of 1/10 (*m*/*v*) sterile PBS, according to Blain et al. [24] with few modifications. The rhizomes and the buffer were ground and mixed using a sterile mortar and pestle. After that, the mixture was serial diluted into dilutions from 10^−1^ to 10^−5^ in sterile distilled water. Then, 500 µL of the dilutions 10^−3^, 10^−4^, and 10^−5^ were plated in triplicate into Petri dishes containing 30 mL of modified PDA or modified MS. Plates were incubated at 25 ± 1 °C until individual colonies or strains were detected. Fungal strains were excised from the original plates and purified. All endophytic microorganisms were stored at 4 °C for further analyses.

Microscope slides were prepared to inspect the morphology of fungal colonization inside *A. uniflora* rhizomes according to Herrera et al. [9] and visualized in a scanning electron microscope SU 3500 (Hitachi, Tokyo, Japan) at a work distance of 5–7.2 mm. 

### 2.3. Molecular Identification of Fungi

Morphological features such as the color and growth rate of the fungal strains were used to classify the isolates and one representative strain per group was selected to perform the molecular identification. DNA from the isolates was extracted from liquid cultures in potato dextrose broth (PDB). The fungi were inoculated in 50 mL Falcon tubes containing 20 mL of PDB and cultured for 10 days in an orbital shaker at 150 rpm and 25 ± 1 °C. Falcon tubes containing fungal mycelia were centrifuged at 10,000 rpm for 5 min and the supernatant was removed. The pellets were washed three times with distilled water, followed by centrifugation at 10,000 rpm for 5 min between each wash. Approximately 100 mg of fungal mycelia were ground to a fine powder using liquid nitrogen and a sterile mortar and pestle. The ground mycelia were subjected to DNA extraction using the DNeasy Plant Mini Kit (Qiagen, Hilden, Germany) according to the manufacturer’s instructions. PCR primers were designed to amplify the internal transcribed spacers by using the ITS1 and ITS4 primers according to White et al. [26]. The PCR cycle consists of an initial denaturing at 95 °C for 5 min, followed by 35 cycles of denaturing at 95 °C for 1 min, annealing at 56 °C for 1 min, extension at 72 °C for 1 min each, and final extension for 5 min at 72 °C. PCR products were checked on 2% agarose gel stained with GelRed^®^ (Biotium Inc., Fremont, CA, USA). Sequencing was performed by Macrogen (Seoul, Korea).

BLAST searches were conducted to find the closest match, accepting the genus and species classification according to Chen et al. [27]. When the isolate matched with *Penicillium* spp. strains, the specific primers Bt2a and Bt2b were used to confirm species assignation, according to Samson et al. [28] and submitted to the GenBank database. To perform estimation of the operational taxonomic units (OTUs), the ITS sequences were aligned using the ClustalW software [29] and the gaps and deletions were eliminated using the BioEdit software [30]. The similarity matrix was obtained in the ClustalW software and the OTUs was assigned by manual comparison at 97% sequence similarity.

### 2.4. Screening of Plant-Growth-Promoting Traits

Phosphate solubilization and production of indole acetic acid (IAA) were estimated according to Ahemad and Khan [31] and Khalid et al. [32], respectively. Additionally, the production of low-molecular-weight organic acids was determined by RP-HPLC, as described in Herrera et al. [33] with modifications. Briefly, liquid cultures from the fungal isolates were performed in PDB media and incubated in an orbital shaker at 140 rpm and 25 ± 1 °C for seven days. The resulting solution was filtered (0.45 μm), freeze-dried, re-suspended in 500 μL deionized sterile water, and filtered again (0.22 μm). Calibration curves were prepared using an organic acids kit (47264, Supelco, Bellefonte, PA, USA). Chromatographic analysis was carried out in a HPLC (Shimadzu CTA-20AC, Kyoto, Japan) equipped with a UV–visible detector. Separation of organic acids was done in a C-18 reverse phase column (MultoHigh 100 RP-18, 5 mm particle size, CS-GmbH, Langerwehe, Germany). The mobile phase was 93% (*v*/*v*) 25 mM KH_2_PO_4_ at pH 2.5 and 7% (*v*/*v*) methanol, with a flow rate of 1 mL min^−1^, according to Cawthray [34].

### 2.5. Data Analyses

For community analyses, data from the frequency of microorganisms from the two sampling points were pooled and analyzed as independent units as described in Koizumi et al. [35]. The frequency of fungal operative taxonomic units (OTUs) in the rhizomes and the relative occurrence were estimated. Furthermore, Shannon’s and Simpson’s species diversities were estimated using EstimateS version 8.2 Xing and Guo [36] and the Margalef’s species richness was calculated according to Yuan et al. [37], by manual calculation. The architecture of the potential mycorrhizal fungi shared between the sampled populations was constructed based on the Fruchterman–Reingold algorithm as defined in Jia et al. [38] using the *R* software (R Core Team 2018; https://www.R-project.org; igraph package [39]).

Quantitative data were analyzed by ANOVA. If the *p* value indicated significant differences between treatments (*p* < 0.05), post hoc pair-wise comparisons were performed, using the SD of means and Tukey’s multiple range test. Statistical significance was set at *p* < 0.05. All statistical tests were conducted using the *R* software (R Core Team 2018; https://www.R-project.org). 

## 3. Results

### 3.1. Sampling

**Figure 1:**
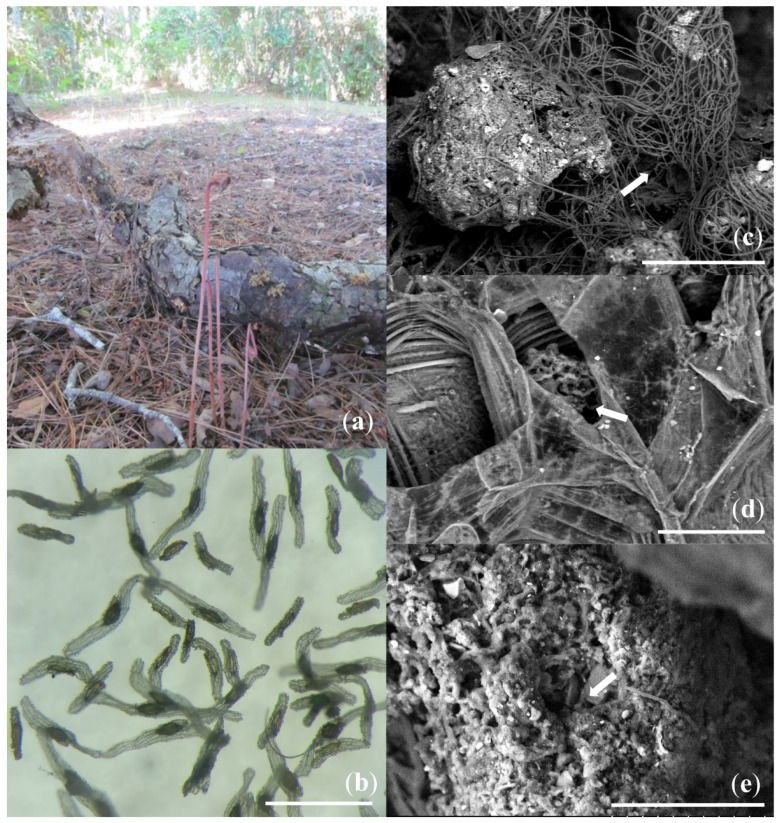
(**a**) *Arachnitis uniflora* growing in the understorey of a mixed forest in Cholchol. (**b**) Dust-like seeds of *A. uniflora* (scale bar = 1 mm). (**c**) Fungal hyphae (white arrow) growing outside the root (scale bar = 100 µm). (**d**) Intracellular hyphal coils (white arrow) inside the root cortex (scale bar = 50 µm). (**e**) Extracellular hyphal coils (white arrow) colonizing the root cortex (scale bar = 50 µm).

Soil chemical characterization, plants, and sporocarp of fungal species from the two sampling sites are described in Table 1. The nitrogen, phosphorous, potassium, and soil organic matter were greater at sampling point 1, which relates with the high diversity of plant species colonizing the sampling site. We showed a different number of *A. uniflora* individuals growing in the two different understoreys. More *A. uniflora* individuals were found at sampling point 1 (27 plants/m^2^) in spite of the populations being smaller (height 14 ± 2.6 cm); whereas at sampling point 2 we showed that *A. uniflora* plants were fewer (12 plants/m^2^) but bigger (height 19 ± 1.5 cm). Sampling point 2 was dominated by *P. radiata* and showed the presence of various ectomycorrhizal fungi, whereas sampling point 1 showed more native plant species and a smaller diversity of ectomycorrhizal fungi (Table 1). The rhizome of the *A. uniflora* plant showed dense external and internal colonization of fungal hyphae, with fungal mycelia outside and inside the root (Figure 1). Hyphal coils, external mycelia, and intracellular colonization were shown, but there was no detection of arbuscules or vesicles inside the analyzed roots (Figure 1). 

### 3.2. Isolation of Fungi

A total of 415 fungal strains were counted, of which 142 and 273 were isolated from the soil of sampling point 1 and 2, respectively (Table 2). We isolated 266 fungal strains from the rhizosphere and 144 fungal strains from the roots (endophytes). Margalef’s richness index and Shannon’s diversity index of the fungal isolates were greater in *A. uniflora* plants from sampling point 1, despite there being more abundance of fungal strains in sampling point 2 (Table 2). After molecular identification based on BLAST alignments, 18 OTUs were defined (Table 3). 

At sampling point 1, ITS sequences of isolates AF01, AF03, AF06, AF07, AF08, AF09, AF10, AF17, and AF21 showed high similarity with different *Penicillium* spp. strains (Table 3). The isolate AF02 showed high similarity with an orchid mycorrhizal fungi isolated from the partially mycoheterotrophic orchid *Limodorum abortivum* and was classified as *Rhizoctonia* sp. Fungal sequences from the isolates AF04, AF22, AF23, and AF24 showed high similarity with potential plant fungal pathogen strains and were classified as *Fusarium* sp., *Phoma* sp., *Paraboeremia* sp., and *Podosphaera* sp., respectively (Table 3). The isolates AF05 and AF11 showed high similarity with the saprophytic strains *Ganoderma australe* and *Trametes versicolor*, respectively (Table 3). *T. versicolor, Penicillium pancosmium* and *Penicillium exsudans* were more frequent in the rhizosphere of the sampling point 1 (frequency of 0.11, 0.08, and 0.09, respectively), whereas *Penicillium montanense*, *Phoma* sp., and *G. australe* were the most frequent endophytic strains (frequency of 0.13, 0.10, and 0.09, respectively) (Table 2).

**Figure 2:**
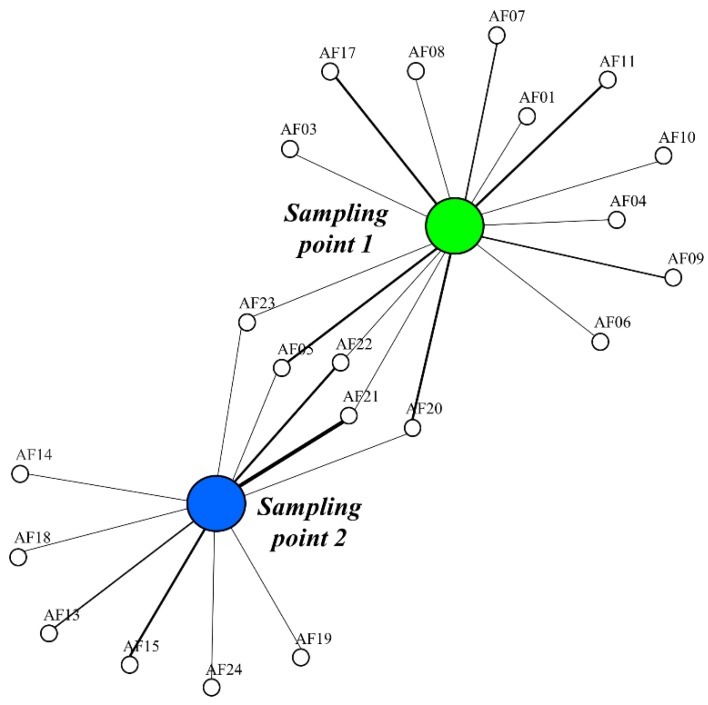
Fungal sharing between *Arachnitis uniflora* plants sampled in two forest understoreys: sampling point 1 (green) and 2 (blue). Line width relates to the isolation frequency of fungi.

At sampling point 2, ITS sequences from isolates AF12, AF13, AF14, AF15, AF16, AF18, AF19, and AF21 showed high similarity with different *Penicillium* spp. strains (Table 3). The isolates AF22, AF23, and AF24 showed high similarity with the potential plant pathogen strains *Phoma* sp., *Paraboeremia* sp., and *Podosphaera* sp., respectively (Table 3). Finally, fungal sequences AF05 and AF20 were classified as *Ganoderma* spp. (Table 3). *Penicillium montanense*, *Penicillium simplicissimum*, and *Penicillium sanguifluum* were more abundant in the rhizosphere of the plants (frequency of 0.19, 0.14, and 0.12, respectively), whereas *Phoma* sp., *P. montanense*, and *Ganoderma annulare* were the most frequent endophytic strains (0.10, 0.08, and 0.06, respectively) (Table 2). The architecture of the root fungal associates was different according to the sampling site. The isolates *G. australe, G. annulare, P. montanense, Phoma* sp., and *Paraboeremia* sp. were the fungi that were isolated under both conditions (Figure 2) (Table 2). All the ITS and beta tubulin fungal sequences obtained are available in the GenBank database under accession numbers MK826009–MK826027 and MN603779–MN603794, respectively.

### 3.3. Production of Plant-Growth-Promoter Metabolites and Organic Acids

Almost no fungal isolates can solubilize phosphate, with the endophytic microorganisms *P. montanense, G. australe*, and *G. annulare* being the microorganisms that showed a weak phosphate solubilization activity (Table 2). The maximum IAA production was found in the fungal isolates *Penicillium brunneoconidiatum* and *Phoma* sp., with 0.44 and 0.25 µg mL^−1^, respectively (Table 2). Siderophore production was detected in five fungal isolates, which showed a solubilization halo of 5.2 mm day^−1^ for *Penicillium exsudans*, 5.7 mm day^−1^ for *P. sanguifluum*, 3.8 mm day^−1^ for *G. australe*, 2.6 mm day^−1^ for *P. brunneoconidiatum*, and 1.6 mm day^−1^ for *G. annulare* (Table 2). 

Regarding organic acid production, our results showed different organic acid production rates, which differed according to the isolation source of the fungi (rhizosphere or endophyte) (Table 2). The highest organic acid production rates of the endophytic isolates were recorded in *Penicillium wollemiicola* (336.9 ± 45.8 and 47.1 ± 8.5 µg mL^−1^ of citric and oxalic acid, respectively), *P. montanense* (1698.3 ± 70.3 and 1084.9 ± 52.8 µg mL^−1^ of lactic and succinic acid, respectively) and *Paraboeremia* sp. (1084.9 ± 52.8 µg mL^−1^ of malic acid) (Table 2). The fungal strains isolated from the rhizosphere showed a greater overall organic acid production rate, especially the isolates *Penicillium panissanguineum* (511.9 ± 11.1 and 1452.2 ± 79.0 µg mL^−1^ of citric and lactic acid, respectively)*, Penicillium asperosporum* (1393.7 ± 150.9 and 1779.2 ± 68.1 µg mL^−1^ of succinic and malic acid, respectively) and *Penicillium roseopurpureum* (137.2 ± 10.0 µg mL^−1^ of oxalic acid) (Table 2)

## 4. Discussion

In our study, we performed an analysis of culturable fungi associated with the mycoheterotrophic plant *A. uniflora* and assessed the potential of rhizosphere and endophytic microorganisms to produce metabolites with a role in promoting plant growth. Previous studies have reported AMF as the main mycorrhizal fungi associated with *A. uniflora* rhizomes, but our analyses did not show the presence of the characteristic arbuscular mycorrhizal structures, which agrees with previous studies analyzing root colonization with molecular methods being necessary to detect and identify the presence of glomalean fungi [17,19,20] (Figure 1). However, our results showed a broader range of culturable fungi associated with the roots, ranging from potential plant pathogens and saprophytic fungi to different endophytic fungi (Table 3). These fungi are common inhabitants of the soils and do not require a live plant to live, as is the case for AMF [52]. Several studies have shown that fully and partially mycoheterotrophic plants depend on mycorrhizal interactions to complement their nutritional demands, and mycoheterotrophy is key to promoting plant development to further developmental stages [8,53]. Our results agree with previous studies analyzing mycorrhizal diversity in orchids, in which diverse pathogenic, ectomycorrhizal, and saprophytic fungi are essential to promoting seed germination and plant development [11,54,55]. These studies showed that free-living fungi are essential in the first developmental stages of the plants, promoting seed germination and the further growth to plantlets in order to complement the nutritional demands of the embryo, which usually lacks sufficient nutritional reserves to start morphogenesis. As orchid seeds, *A. uniflora* produce hundreds of dust-like seeds (Figure 1), which require the external nutritional supply of carbon and mineral nutrients by mycoheterotrophy. At this life stage is when free-living fungi, such as the endophytic fungi isolated in our study, can be useful to start seed germination and promote the first developmental steps, prior to mycorrhization with other fungi, as is reported in the orchid *Goodyera pubescens* [56], and some partially mycoheterotrophic orchids from Chile [57]. 

The mycorrhizal association of fully or partially mycoheterotrophic plants is influenced by the ecosystem in which the plant grows [58,59]. Domínguez et al. [16] showed that the rhizome of *A. uniflora* can host various fungal propagules of AMF and that this kind of mycorrhizal association is the main one in flowering plants. However, our image analyses did not show any signs of arbuscular mycorrhizal colonization. In fact, we found symbiotic structures similar to those reported for ectomycorrhizal and ectendomycorrhizal associations, with several hyphae outside the root cortex and a strong presence of intraradical colonization (Figure 1) [52,60,61], which agree with Domínguez et al. [20] who showed that intraradical fungal colonization is morphologically different to what is reported in arbuscular mycorrhizal plants. Moreover, diverse hyphal coils were noted, similarly to the peloton-like structures reported in orchid mycorrhizal associations, where the mycorrhizal fungi enter the root cortex and form hyphal coils as an interchangeable surface for nutrients and water [62,63,64]. Even fully mycoheterotrophic orchids can host ectomycorrhizal and pathogenic fungi inside hyphal coils, suggesting that intraradical colonization can be different according to the plant [54,65,66]. Although we did not show the typical arbuscular mycorrhizal structures inside the rhizomes, we do not rule out the mycorrhizal association with AMF, particularly at further developmental stages where the nutritional requirements are greater and the plant can produce metabolites with a key role in fungal attraction. The microhabitats in which *A. uniflora* grows showed different native plant species, and mixed with different exotic plants (Table 1). This change in mycorrhizal associations has been reported for several plants that show different mycorrhizal preferences according to the distribution range, physiology state, and age of the plants [59,67,68,69]. Our results agree with Roy et al. [70] and Martos et al. [71], who have described saprophytic and litter-decaying fungi associated with the achlorophyllous plants *Neottia* spp. and *Wullschlaegelia aphylla*, respectively.

Fungal associations are essential to establishing webs between adjacent plants through fungal mycelia, and these processes are essential for non-photosynthetic plants such as *A. uniflora* [11,72]. Recent studies have demonstrated common mycorrhizal networks in which a single fungus can simultaneously associate with unrelated plants, which usually leads to a better plant response to pathogenic fungi, increased growth and carbon transfer, increased photosynthesis, increased uptake of soil nutrients, and tolerance to stress, among others [73,74,75]. Furthermore, these processes have been assessed in several mycoheterotrophic plants, in which the mycorrhizal hyphae growing outside the roots are essential to complement plant nutrition [76]. Our study showed saprophytic fungi associated with *A. uniflora* roots, specifically in individuals sampled at sampling point 2, where several fungi are characteristic of the decaying wood present in the soils. Even though the sampling sites were close to each other, we found a high variation in the fungi associated with the *A. uniflora* plant. These processes of changing mycorrhizal patterns under different ecosystems have been reported in several orchids [56,67]

*A. uniflora* plants produce thousands of minute seeds in a single capsule. These seeds are characterized by a poorly developed endosperm with minimal nutritional reserves for the embryo (Figure 1). A huge number of plants depend on mycorrhizal fungi to compensate for this lack of nutritional reserves for the embryo after an initial infection with a compatible symbiont, which acts as the main nutritional source for the plant [8,77]. We think that the association with free-living fungi is the way that *A. uniflora* plants have come to compensate for the poor endosperm, in which mycoheterotrophy is essential in the first developmental stage. Thus, we suggest that a fungal switch may be determinant in the life cycle of the plant, in which AMF may play a crucial role in the adaptation of the plant to pristine ecosystems, which can be essential at further developmental stages as reported by Domínguez et al. [20] and Renny et al. [19]. These are the main mechanisms developed by various non-photosynthetic orchids, in which the association with diverse ectomycorrhizal and saprophytic fungi is necessary throughout life [54,78,79].

Endophytic fungi usually have a positive effect on plant communities, increasing the plant’s fitness by conferring abiotic and biotic stress tolerance, increasing biomass, plant growth, and yield by increasing nutrient uptake or suppressing pathogen via antifungal activity [80]. Our results suggest that some of the fungal strains can be considered beneficial endophytes due to their capacity for phosphate solubilization, IAA production, siderophore production, and organic acid exudation, which can help the plant complement its nutritional demands. Our results agree with several studies identifying endophytic fungi as potential plant-growth-promoter microorganisms, as has been reported for cucumber plants treated with *Penicillium* sp. and *Phoma* sp., as these improve the plant’s defense against disease [81,82,83]. Furthermore, several *Penicillium* spp. have been isolated from the plant’s phyllosphere, rhizosphere, and endosphere, as well as from different decaying fruits [84]. Similarly, Soltani and Moghaddam [85] identified *Phoma* spp. and *Penicillium* sp. strains associated with a *C. sempervirens* forest which agree with our results with respect to *Phoma* sp. in the *A. uniflora* roots from sampling point 2, where *C. sempervirens* was the most abundant species. Additionally, in the roots sampled at sampling point 1 we isolated a fungal strain that matched with *Rhizoctonia* sp., an orchid mycorrhizal fungus associated with *Limodorum abortivum*, which is often isolated from cortical cells of partially mycoheterotrophic orchids [86,87]. This finding may underline a critical step at the seed germination stage, but these processes must be addressed in further studies. Despite some of the fungal genera isolated in our study being considered potential plant pathogen strains, the ability of mycoheterotrophic plants to revert the pathogenicity of fungal strains must be considered, particularly if several fungal strains with a protective role are accepted as endophytes, similar to what has been reported for *Penicillium janthinellum* isolated from the leaves of *Panax notoginseng* [88]. 

Key aspects of the life cycle of *A. uniflora* remain unclear, especially at seed germination and the time in which AMF begins to be essential for the plant [18]. Considering that colonization by a compatible mycorrhizal fungi is needed for early development in all mycoheterotrophic plants [1], it is necessary to know which fungi are necessary to start and maintain the developmental processes. The association of the minute seeds with different culturable fungi could represent an opportunity to study the ability of the isolated strains to promote seed germination in artificial media in order to explore more about the molecular regulations of this plant at the first developmental stages. In vitro seed germination of non-photosynthetic plants using compatible mycorrhizal fungi has been achieved in several species, as in the case of the achlorophyllous plants *Neottia nidus-avis* and *Gastrodia elata* [89,90]. Further studies must address the potential of the endophytic microorganisms isolated in our study to promote seed germination to later developmental stages.

## 5. Conclusions

Endophytic microorganisms are beneficial to plant physiology by producing different metabolites that can improve growth and fitness in diverse environments. Additionally, the root associations of mycoheterotrophic plants with diverse endophytic fungi could be a crucial step for ecological applications. Our study contributes to understanding the adaptation of *A. uniflora* to different ecological conditions and describes different interactions with free-living fungi under two different ecosystems in southern Chile. These culturable microorganisms could be considered candidates to study the effects of fungi in seed germination and development of this fully mycoheterotrophic plant. Further studies must address the potential of free-living fungi to promote seed germination under axenic conditions, a previous step to evaluating the molecular mechanisms involved in the life cycle of this mycoheterotrophic plant.

## Figures and Tables

**Table 1:** Soil chemical characterization, plant species, and fungal sporocarps in the two microhabitats with *Arachnitis uniflora* plants.

	Sampling Point 1	Sampling Point 2
**Soil chemical** **Characterization**		
N ^a^	52	28
P ^a^	19	11
K ^a^	489	293
pH ^b^	6.18	5.51
Organic matter ^c^	41	29
K ^d^	1.25	0.75
Na ^d^	0.15	0.15
Ca ^d^	27.70	9.23
Mg ^d^	9.50	3.63
Al ^d^	0.01	0.27
CEC ^d^	38.61	14.03
**Plant species** **^e^**	*Eucryphia cordifolia* (Ulmo) *Nothofagus obliqua* (Roble) *Cryptocarya alba* (Peumo) *Peumus boldus* (Boldo) *Lapageria rosea* (Copihue) *Aristotelia chilensis* (Maqui) *Sophora cassioides* (Pelu) *Lomatia hirsuta* (Radal) *Chusquea quila* (Quila) *Persea lingue* (Lingue) *Drimys winteri* (Canelo) *Luma apiculata* (Arrayan) *Gevuina avellana* (Avellano) *Chloraea galeata* *Chloraea philippi*	*Cupressus sempervirens* (Cypress) *Pinus radiata* (Pino) *Peumus boldus* (Boldo) *Luma apiculata* (Arrayan) *Gavilea araucana*
**Fungal sporocarps**	*Morchella esculenta* *Bondarzewia guaitecasensis* *Cortinarius magellanicus* *Ramaria flaccida* *Ramaria stricta* *Russula nothofaginea*	*Lactarius deliciosus* *Suillus luteus* *Aleuria aurantia* *Amanita muscaria* *Amanita rubescens* *Mycena alcalina* *Russula sardonia* *Mycena leptocephala* *Mycena chusqueophila*

^a^ mg kg^−1^ (total contents); ^b^ In H_2_O; ^c^ %; ^d^ (meq/100 g); ^e^ Scientific name (common name).

**Table 2:** Screening of potential plant-growth-promoting traits and abundance of rhizospheric and endophytic fungi isolated from *Arachnitis uniflora* roots from the Coastal Mountains in southern Chile. Results are means ± standard error (*n* = 5). Different letters indicate significant differences between treatments according to Tukey’s multiple range test (*p* < 0.05).

Fungal Isolate	Sampling Point 1 (frequency)	Sampling Point 2 (frequency)	IAA (µg mL^−1^)	Phosphate Solubilization	Siderophore Production	Citric Acid (µg mL^−1^)	Lactic Acid (µg mL^−1^)	Succinic Acid (µg mL^−1^)	Malic Acid (µg mL^−1^)	Oxalic Acid (µg mL^−1^)
**AF01**	5 (0.03)	-	0.14 ^cd^	-	-	336.9 ± 45.8 ^b^	412.2 ± 152.2 ^efgh^	151.6 ± 20.4 ^e^	476.7 ± 87.4 ^d^	47.1 ± 8.5 ^c^
**AF02**	2 (0.01)	-	0.02 ^g^	-	-	53.9 ± 15.6 ^fg^	407.2 ± 99.3 ^fgh^	459.2 ± 48.9 ^c^	918.1 ± 263.4 ^c^	-
**AF03**	5 (0.04)	-	0.19 ^c^	-	-	-	-	-	-	4.0 ± 1.9 ^e^
**AF04**	12 (0.08)	-	0.12 ^de^	-	-	187.9 ± 10.2 ^cde^	99.5 ± 13.1 ^ij^	68.4 ± 22.3 ^e^	105.1 ± 13.6 ^de^	-
**AF05**	15 (0.09)	3 (0.01)	0.05 ^fg^	+	++	190.8 ± 8.5 ^cde^	378.2 ± 51.4 ^ghi^	575.8 ± 68.3 ^c^	39.8 ± 10.2 ^e^	44.8 ± 7.3 ^cd^
**AF06**	3 (0.02)	-	-	-	-	130.3 ± 17.1 ^defg^	705.6 ± 43.9 ^def^	1393.7 ± 150.9 ^a^	1779.2 ± 68.1 ^a^	20.6 ± 4.5 ^de^
**AF07**	11 (0.08)	-	0.04 ^fg^	-	-	244.8 ± 63.4 ^bcd^	124.0 ± 21.9 ^hij^	198.5 ± 33.5 ^de^	-	-
**AF08**	8 (0.06)	-	0.03 ^fg^	-	-	126.8 ± 20.8 ^efg^	142.6 ± 17.6 ^hij^	96.3 ± 6.1 ^e^	1258.8 ± 123.4 ^bc^	-
**AF09**	10 (0.08)	-	-	-	++	35.4 ± 6.7 ^g^	1005.7 ± 95.9 ^cd^	21.8 ± 1.6 ^e^	-	-
**AF10**	7 (0.05)	-	0.08 ^efg^	-	-	160.2 ± 17.0 ^cdef^	-	122.1 ± 4.2 ^e^	23.9 ± 2.8 ^e^	-
**AF11**	16 (0.11)	-	-	-	-	-	49.0 ± 4.8 ^j^	-	-	41.0 ± 5.7 ^cd^
**AF12**	-	32 (0.12)	0.06 ^fg^	-	++	-	189.2 ± 12.9 ^hij^	31.9 ± 3.4 ^e^	-	14.1 ± 6.6 ^e^
**AF13**	-	24 (0.09)	-	-	-	-	6.0 ± 0.9 ^j^	-	1565.7 ± 113.6 ^ab^	-
**AF14**	-	22 (0.08)	0.04 ^fg^	-	-	320.2 ± 12.5 ^b^	1203.9 ± 43.1 ^bc^	384.3 ± 12.5 ^cd^	383.6 ± 34.5 ^de^	137.2 ± 10.0 ^a^
**AF15**	-	39 (0.14)	0.04 ^fg^	-	+	-	10.2 ± 1.4 ^j^	-	-	-
**AF16**	-	51 (0.19)	0.02 ^g^	+	-	138.8 ± 10.8 ^defg^	494.4 ± 25.9 ^efg^	207.4 ± 11.8 ^de^	10.8 ± 1.7 ^e^	3.4 ± 0.3 ^e^
**AF17**	18 (0.13)	-	-	+	-	-	-	9.2 ± 0.5 ^e^	-	3.4 ± 0.5 ^e^
**AF18**	-	16 (0.06)	0.44 ^a^	+	+	103.2 ± 4.3 ^efg^	711.1 ± 40.0 ^de^	47.8 ± 1.7 ^e^	386.9 ± 17.6 ^de^	103.8 ± 6.2 ^b^
**AF19**	-	9 (0.03)	0.13 ^d^	-	-	511.9 ± 11.1 ^a^	1452.2 ± 79.0 ^ab^	-	262.5 ± 7.4 ^de^	4.9 ± 0.3 ^e^
**AF20**	7 (0.05)	15 (0.06)	0.14 ^d^	+	-	266.1 ± 13.9 ^bc^	713.9 ± 23.2 ^de^	147.7 ± 15.8 ^e^	22.8 ± 1.6 ^e^	-
**AF21**	7 (0.05)	21 (0.08)	0.06 ^fg^	+	-	128.7 ± 15.8 ^efg^	1698.3 ± 70.3^a^	1084.9 ± 52.8^b^	22.3 ± 0.3 ^e^	2.6 ± 0.4 ^e^
**AF22**	14 (0.10)	26 (0.10)	0.25 ^b^	-	-	180.9 ± 9.2 ^cde^	-	160.5 ± 19.4^de^	9.3 ± 0.4 ^e^	-
**AF23**	2 (0.02)	7 (0.02)	0.08 ^ef^	-	-	40.2 ± 2.1 ^g^	516.3 ± 13.1^efg^	1016.8 ± 24.7^b^	1293.8 ± 53.0 ^bc^	3.0 ± 0.5 ^e^
**AF24**	-	8 (0.03)	-	-	-	-	-	-	-	-
**Total strains**	142	273								
**Margalef’s richness index**	2.845	2.136								
**Shannon’s species diversity**	3.721	3.413								
**Simpson’s species diversity**	0.076	0.107								

**Table 3:** Molecular identification of culturable fungi isolated from *Arachnitis uniflora* roots in southern Chile, based on the closest match in the GenBank database. GenBank accession numbers in bold are the sequences obtained with the ITS primers, whereas italic accessions are from the beta tubulin sequences.

Fungal Isolate *Final Identification*	GenBank Accession Numbers	Isolation Source	Close Relatives (Accession Number)	% Identity	Reference
**AF01** *Penicillium wollemiicola*	**MK826009** *MN603790*	Endophyte (Sampling point 1)	*Penicillium wollemiicola* (KJ174314)	99	Visagie et al. [40]
**AF02** *Rhizoctonia* sp.	**MK826027**	Endophyte (Sampling point 1)	*Rhizoctonia* sp. (DQ061931)	99	Girlanda et al. [41]
**AF03** *Penicillium spinulosum*	**MK826010** *MN603791*	Endophyte (Sampling point 1)	*Penicillium spinulosum* (KT316692)	99	Vu et al. [42]
**AF04** *Fusarium* sp.	**MK826011**	Endophyte (Sampling point 1)	*Fusarium oxysporum* (GQ121287)	100	GenBank
**AF05** *Ganoderma australe*	**MK826012**	Endophyte (Sampling point 1 and 2)	*Ganoderma australe* (KU569541)	100	Bolaños et al. [43]
**AF06** *Penicillium asperosporum*	**MK826026** *MN603792*	Rhizosphere (Sampling point 1)	*Penicillium asperosporum* (JN376151)	99	You et al. [44]
**AF07** *Penicillium pancosmium*	**MK826013** *MN603789*	Rhizosphere (Sampling point 1)	*Penicillium pancosmium* (MF803943)	100	Visagie et al. [45]
**AF08** *Penicillium miczynskii*	**MK826014** *MN603786*	Rhizosphere (Sampling point 1)	*Penicillium miczynskii* (MH865287)	99	Vu et al. [42]
**AF09** *Penicillium exsudans*	**MK826015** *MN603793*	Rhizosphere (Sampling point 1)	*Penicillium exsudans* (MH864309)	99	Vu et al. [42]
**AF10** *Penicillium sanguifluum*	**MK826016** *MN603787*	Rhizosphere (Sampling point 1)	*Penicillium sanguifluum* (MH858377)	99	Vu et al. [42]
**AF11** *Trametes versicolor*	**MK826025**	Rhizosphere (Sampling point 1)	*Trametes versicolor* (KY824790)	99	GenBank
**AF12** *Penicillium sanguifluum*	**MK826017** *MN603788*	Rhizosphere (Sampling point 2)	*Penicillium sanguifluum* (MH858377)	99	Vu et al. [42]
**AF13** *Penicillium* sp.	**MK826018** *MN603794*	Rhizosphere (Sampling point 2)	*Penicillium* sp. (KY401069)	99	Vera et al. [46]
**AF14** *Penicillium roseopurpureum*	**MK826019** *MN603779*	Rhizosphere (Sampling point 2)	*Penicillium roseopurpureum* (MH865745)	99	Vu et al. [42]
**AF15** *Penicillium simplicissimum*	**MK826020** *MN603780*	Rhizosphere (Sampling point 2)	*Penicillium simplicissimum* (KM458844)	99	GenBank
**AF16** *Penicillium montanense*	**MK826021** *MN603781*	Rhizosphere (Sampling point 2)	*Penicillium montanense* (HQ157959)	99	Kernaghan and Patriquin [47]
**AF17** *Penicillium montanense*	**MK826022** *MN603783*	Endophyte (Sampling point 1)	*Penicillium montanense* (HQ157959)	99	Kernaghan and Patriquin [47]
**AF18** *Penicillium brunneoconidiatum*	**MK826007** *MN603784*	Rhizosphere (Sampling point 2)	*Penicillium brunneoconidiatum* (JX140766)	100	Visagie et al. [48]
**AF19** *Penicillium* *panissanguineum*	**MK826008** *MN603785*	Rhizosphere (Sampling point 2)	*Penicillium panissanguineum* (KT887862)	99	Visagie et al. [49]
**AF20** *Ganoderma annulare*	**MK826006**	Endophyte (Sampling point 1 and 2)	*Ganoderma annulare* (JQ520160)	100	Park et al. [50]
**AF21** *Penicillium montanense*	**MK826005** *MN603782*	Endophyte (Sampling point 1 and 2)	*Penicillium montanense* (HQ637347)	100	Zhou et al. [51]
**AF22** *Phoma* sp.	**MK826024**	Endophyte (Sampling point 1 and 2)	*Phoma herbarum* (KY979198)	100	GenBank
**AF23** *Paraboeremia* sp.	**MK826004**	Endophyte (Sampling point 1 and 2)	*Paraboeremia putaminum* (MH858878)	100	Vu et al. [42]
**AF24** *Podosphaera* sp.	**MK826023**	Endophyte (Sampling point 2)	*Podosphaera* sp. (EU273519)	100	GenBank
